# Phenotypic Characterisation of *Shewanella oneidensis* MR-1 Exposed to X-Radiation

**DOI:** 10.1371/journal.pone.0131249

**Published:** 2015-06-22

**Authors:** Ashley R. Brown, Elon Correa, Yun Xu, Najla AlMasoud, Simon M. Pimblott, Royston Goodacre, Jonathan R. Lloyd

**Affiliations:** 1 Williamson Research Centre for Molecular Environmental Science, University of Manchester, Oxford Road, Manchester, M13 9PL, United Kingdom; 2 Research Centre for Radwaste and Decommissioning, University of Manchester, Oxford Road, Manchester, M13 9PL, United Kingdom; 3 School of Earth, Atmospheric and Environmental Sciences, University of Manchester, Oxford Road, Manchester, M13 9PL, United Kingdom; 4 Manchester Institute of Biotechnology, University of Manchester, 131 Princess Street, Manchester, M1 7DN, United Kingdom; 5 Dalton Cumbrian Facility, University of Manchester, Westlakes Science and Technology Park, Moor Row, Cumbria, CA24 3HA, United Kingdom; 6 School of Chemistry, University of Manchester, Oxford Road, Manchester, M13 9PL, United Kingdom; The University of Akron, UNITED STATES

## Abstract

Biogeochemical processes mediated by Fe(III)-reducing bacteria such as *Shewanella oneidensis* have the potential to influence the post-closure evolution of a geological disposal facility for radioactive wastes and to affect the solubility of some radionuclides. Furthermore, their potential to reduce both Fe(III) and radionuclides can be harnessed for the bioremediation of radionuclide-contaminated land. As some such sites are likely to have significant radiation fluxes, there is a need to characterise the impact of radiation stress on such microorganisms. There have, however, been few global cell analyses on the impact of ionizing radiation on subsurface bacteria, so here we address the metabolic response of *S*. *oneidensis* MR-1 to acute doses of X-radiation. UV/Vis spectroscopy and CFU counts showed that although X-radiation decreased initial viability and extended the lag phase of batch cultures, final biomass yields remained unchanged. FT-IR spectroscopy of whole cells indicated an increase in lipid associated vibrations and decreases in vibrations tentatively assigned to nucleic acids, phosphate, saccharides and amines. MALDI-TOF-MS detected an increase in total protein expression in cultures exposed to 12 Gy. At 95 Gy, a decrease in total protein levels was generally observed, although an increase in a putative cold shock protein was observed, which may be related to the radiation stress response of this organism. Multivariate statistical analyses applied to these FT-IR and MALDI-TOF-MS spectral data suggested that an irradiated phenotype developed throughout subsequent generations. This study suggests that significant alteration to the metabolism of *S*. *oneidensis* MR-1 is incurred as a result of X-irradiation and that dose dependent changes to specific biomolecules characterise this response. Irradiated *S*. *oneidensis* also displayed enhanced levels of poorly crystalline Fe(III) oxide reduction, though the mechanism underpinning this phenomenon is unclear.

## Introduction

Civil nuclear energy generation and nuclear weapon production since 1945 has generated significant volumes of legacy radioactive wastes and contaminated land [1]. As physicochemical methods of remediation of contaminated land, e.g. soil washing and ‘pump and treat’, may incur great cost, the use of non-invasive *in situ* alternative technologies, such as bioremediation, may provide a more versatile and cost-effective substitute [2,3].

Many subsurface bacteria, such as *Shewanella* spp. have the ability to couple the oxidation of organic matter to the reduction of a range of metal cations, anions and radionuclides [4–6], thus providing the potential for use in the bioremediation of radionuclide contaminated land [2,7]. For example, the precipitation of mobile soluble species such as Tc(VII) and U(VI) can be achieved by their reduction to insoluble Tc(IV) and U(IV) [8,9]. However, as many of the sites contaminated by radionuclides are likely to have significant radiation fluxes [10–12], the utility of microorganisms in the remediation of highly radioactive wastes will largely be determined by the ability to survive radiation stress [13].

Furthermore, microorganisms can potentially affect some processes pertinent to the post-closure evolution of a geological disposal facility. For instance, microbial activity may play an important role in the generation and consumption of gases, such as methane and hydrogen, from the degradation and corrosion of waste. Microorganisms can also control radionuclide speciation and mobility and the biodegradation of potential radionuclide complexants [7,14,15]. Microbial Fe(III) reduction is of particular interest in these environments as the mobility of redox-active radionuclides may be restricted via their reduction by biogenic Fe(II)-bearing phases [7,16]. However, as noted before, the organisms promoting these processes may be subject to significant radiation doses. For example, some predictions of dose rates at waste canister surfaces and in backfill material have been as high as 52 Gy h^-1^ and 72 Gy h^-1^ [17,18]. Consequently, radiation toxicity may govern the importance of microbially controlled processes in these environments and hence, there is a requirement to deliver fundamental physiological information on the impact of ionizing radiation on Fe(III)-reducing bacteria such as *Shewanella oneidensis*.

Many early studies of radiation sensitivity considered DNA as the principal target of radiation due to modification of DNA by both direct (energy deposition into the molecule from the radiation track) and indirect (reaction with reactive oxygen species from the radiolysis of water) mechanisms [19]. Hence, it was thought that the biological impacts of ionizing radiation arose from DNA damage [20] and thus damage to DNA has become a hallmark indicator of the effects of ionizing radiation [21].

*Shewanella* spp. sustain a similar amount of damage to DNA as many other species, yet are considerably more sensitive to radiation than *Escherichia coli* or *Deinococcus radiodurans* [19,22]. Furthermore, the genome of *S*. *oneidensis* MR-1 encodes a conventional set of DNA repair mechanisms which are strongly induced after irradiation [23]. This paradox suggests that the impact of ionizing radiation is more complex than just DNA damage and the cellular response may in fact arise from a large array of potential cellular targets [23]. For instance, it has been reported that proteins are likely the initial target of damage by reactive oxygen species [24] and protein oxidation has been quantifiably related to bacterial sensitivity to ionizing radiation [21,25].

In addition to proteins, ionizing radiation has also been shown to damage lipids, for example via fragmentation and peroxidation [26]. As lipids and lipoproteins are major components of biological membranes, radiation induced reactions to these molecules could alter the integrity and function of membranes, for instance, by changes in viscosity and permeability [27]. Indeed, membrane composition and fluidity may be important to radiation sensitivity where DNA is associated with the membrane [28]. In the case of *S*. *oneidensis*, many of the respiratory cytochromes and respiratory chain components are associated with the outer membrane [29] and thus, oxidative damage to the membrane could restrict this species’ ability to use alternative electron acceptors, such as Fe(III) or radionuclides, whilst not necessarily being lethal. In fact, the *c*-type cytochromes themselves may be a significant source of superoxide, which in turn may yield a suite of reactive oxygen species [30]. It has therefore been proposed that energy metabolism during recovery of *S*. *oneidensis* from irradiation could underpin its sensitivity [22]. Thus, paradoxically, whilst many cytochromes give *S*. *oneidensis* respiratory versatility, this may predispose its metabolism to oxidative stress and sensitivity to radiation [19].

It is evident that the mechanisms of cell killing by ionizing radiation are complex and remain poorly defined. Many previous studies have tried to identify the molecular targets which result in radiation sensitivity; however, there is a paucity of information regarding the whole cell response. A global cell analysis of the impact of a range of doses would allow an assessment of whether the phenotypic response to radiation is predictable. Furthermore, quantification of changes to the levels of specific biomolecules would provide useful information required to assess the physiological status and metabolic capabilities of irradiated *S*. *oneidensis*.

Here, we profile the whole cell metabolism of *S*. *oneidensis* MR-1 exposed to acute X-radiation doses (12 to 95 Gy) via Fourier transform infrared (FT-IR) spectroscopy and matrix-assisted laser desorption/ionization time-of-flight mass spectrometry (MALDI-TOF-MS). Whilst exposure of organisms to radiation fluxes during bioremediation applications and geodisposal scenarios will likely be persistent rather than transient, the acute doses used in this study allow for fundamental changes in metabolism to be probed. Indeed, doubling times in the subsurface are typically large and, as such, populations will be exposed to radiation doses whilst growth rates are very low as is simulated in this study. In addition, the application of an acute dose allows metabolism to be assessed throughout a simulated ‘recovery period’, i.e. after significant radioactive decay has occurred in the subsurface. We chose FT-IR spectroscopy as it allows rapid, quantitative differentiation of metabolic changes in bacteria, through a phenotypic spectroscopic fingerprint, with minimal sample preparation [31], allowing us to report both whole cell metabolism of irradiated cultures and specific changes to biomolecules which underpin the phenotypic response of this organism to radiation.

## Materials and Methods

### Growth of cells

All cultures of *S*. *oneidensis* MR-1 were grown aerobically in a fully defined, pre-sterilized, liquid minimal medium (pH 7.4) based on that described previously by Myers and Nealson [32]: 9 mM (NH_4_)_2_SO_4_; 5.7 mM K_2_HPO_4_; 3.3 mM KH_2_PO_4_; 2.2 mM NaHCO_3_; 1 mM MgSO_4_·7H_2_O; 0.49 mM CaCl_2_·2H_2_O; 67.2 μM Na_2_EDTA; 56.6 μM H_3_BO_3_; 10 μM NaCl; 5.4 μM FeSO_4_·7H_2_O; 5 μM CoCl_2_·6H_2_O; 5 μM NiCl_2_·6H_2_O; 3.9 μM Na_2_MoO_4_·2H_2_O; 1.5 μM Na_2_SeO_4_; 1.3 μM MnCO_3_; 1 μM ZnCl_2_; 0.2 μM CuSO_4_·5H_2_O; 20 mg L^-1^ L-arginine HCl; 20 mg L^-1^ L-glutamate; 20 mg L^-1^ L-serine; 100 mM sodium DL-lactate (carbon source and electron donor); 20 mM fumarate (electron acceptor). Six sterile Erlenmeyer flasks containing the defined aerobic medium were then inoculated with a late log/early stationary phase culture to give an optical density of 0.2 at 600 nm (approximately 5×10^8^ cells mL^-1^).

### Irradiations

Half of the inocula were irradiated at ambient room temperature to a dose of 12 Gy using a Faxitron CP-160 Cabinet X-radiator (160kV; 6 mA; tungsten target). It should be noted that X-radiation is a good analogue for gamma radiation, offering “hard” X-rays with energy comparable to that of photons at the longer wavelength of the gamma spectrum. The remaining 3 replicates formed the non-irradiated control cultures and were lead shielded inside the irradiator to provide controls for any potential unavoidable temperature changes (e.g. 120 min at 20 to 30°C for the 95 Gy treatment). After irradiation, all cultures were incubated at 30°C and shaken at 130 rpm. This procedure and subsequent analysis was repeated with batch cultures for the range of doses: 12, 24, 48, 72 and 95 Gy. Prior to irradiation, the dose rate was determined as 0.79 Gy min^-1^ using Fricke dosimetry as described previously [33–35].

### Quantification of growth

Biomass in each biological replicate was determined at regular time intervals with 1 mL samples extracted for analysis of optical density at 600 nm (OD_600_) using a UV/Vis spectrophotometer (Jenway, UK).

### Viability

Cell viability in all replicates was determined immediately after irradiation via serial dilution in phosphate buffered saline (PBS) solution and subsequent plating on to minimal medium agar plates followed by incubation for two days at 30°C. The number of colony forming units (CFUs) was then determined over the dose range.

### Analysis of metabolism by FT-IR spectroscopy

The metabolic fingerprints of control and irradiated cells were recorded by FT-IR spectroscopy. Aliquots from each biological replicate were collected immediately after irradiation (lag phase), during mid exponential phase and at the maximum biomass yield of stationary phase. Samples were then centrifuged at 4°C at 12,000 *g* for 20 min after which the supernatant was removed and the cell pellet was washed twice with sterile 0.9% NaCl solution prior to being stored at -80°C. Upon analysis, samples were thawed and resuspended into three separate samples in sterile 0.9% NaCl solution to an OD_600_ of 5. A 96 well Si sample plate was washed thoroughly with 2-propanol and deionized water and allowed to dry at room temperature prior to use. 20 μL of each bacterial sample was then applied evenly in triplicate onto the plate (so called technical replicates) prior to drying at 55°C in an oven for 10 min. All FT-IR spectroscopy analysis was conducted using an Equinox 55 infrared spectrometer equipped with a high throughput motorized microplate module, HTS-XT (Bruker Optics, Coventry, UK) as described elsewhere [36]. A deuterated triglycine sulfate (DTGS) detector was employed for absorbance measurements of the samples to be acquired. Thus, 9 spectra from each biological repeat were collected over the wavelength range of 4000 to 600 cm^-1^ using the Opus software (Bruker Optics). Spectra were acquired at a resolution of 4 cm^-1^ with 64 spectra co-added and averaged to improve the signal-to-noise ratio. The collection time for each spectrum was approximately 1 min.

### Data processing

The ASCII data files were imported into MATLAB 2008a (The MathWorks Inc., Natwick, US). FT-IR spectra were normalized using extended multiplicative signal correction (EMSC) [37] and signals arising from CO_2_ in the regions 2400–2275 cm^-1^ and <700 cm^-1^ were removed from the spectra and filled with a smoothed trend [36] before being exported to R version 2.9.2 for further analysis (R Foundation for Statistical Computing, Vienna, Austria). Prior to multivariate statistical analyses, the data were autoscaled by transforming the intensities for each wavenumber such that the mean was equal to zero and the standard deviation equal to 1 [38].

### Partial least-squares analysis

Supervised multivariate classification of treatments using partial least-squares (PLS) regression was used to model the relationship between dose and metabolic fingerprints for all growth phases. The model was calibrated with FT-IR spectra from a “training” data set and used to predict the known doses on a separate “test” set. The objective of the PLS model is to predict the classification of the new samples and in doing so, any separation between classes is displayed in score plots of the principal components [39]. Each model was cross-validated and an *R* ^2^ value generated, as described by Correa et al. [40], in order to measure prediction performance.

### Discriminant analysis

Prior to discriminant function analysis (DFA), principal component analysis (PCA) was used to reduce the dimensionality of the FT-IR data from multiple absorbance measurements down to the first 5 principal components (PCs). The unsupervised method was performed on FT-IR data from all growth phases at each dose, such that the 5 PCs extracted represented the following percentages of the total variance in the FT-IR spectra of each treatment: 12 Gy = 96%; 24 Gy = 99%; 48 Gy = 98%; 72 Gy = 95%; 95 Gy = 97%. The PCs extracted were then used for DFA (and the combined method may be referred to as PC-DFA), as described previously [41,42]. DFA is a supervised method, whereby discrimination between groups is based on *a priori* knowledge of the experimental class structure. The algorithm finds linear combinations of the variables (canonical variates of the PCs fed into the algorithm) which maximize the ratio of between-group variance to within-group variance [40,43]. In order to quantify separation between control samples and treated samples at each growth phase, Euclidean distances between group centres were calculated from the scores plots of the first 3 DFs.

### Quantification of spectral peak areas

As shall be discussed below, PLS and PC-DFA revealed a number of significant peaks that changed with respect to X-radiation dose. Therefore, the peak integrals and peak intensities of FT-IR spectral features were quantified by subtracting a linear base line trend from each individual peak region and then calculating the peak area and maximum absorbance of the peak area (performed in R version 2.9.2; R foundation for Statistical Computing, Vienna, Austria). FT-IR peak regions included the amide I/II region between 1479 to 1775 cm^-1^, the CH_2_/CH_3_ asymmetric stretch at 2885 to 2945 cm^-1^ and aliphatic CH vibrations between 2949 and 2993 cm^-1^. The region from 988 to 1187 cm^-1^ was also consistently highlighted in the loadings extracted during PCA and PLS analysis and maximum peak intensities were also calculated here, with wavenumber assignments discussed below. In addition, absorbance in the nucleic acid and carboxylic acid regions of 860 to 980 cm^-1^ was also assessed, with intensities calculated at 915 cm^-1^.

### MALDI-TOF-MS

Quantification of proteins in irradiated *S*. *oneidensis* MR-1 was achieved using matrix-assisted laser desorption/ionization mass spectrometry (MALDI-MS). As for FT-IR spectroscopy, an aliquot was collected from each biological replicate at lag phase (immediately after irradiation), exponential phase and stationary phase. Prior to analysis, samples were washed twice in sterile deionized water and resuspended in 50 μL of deionized water to an OD_600nm_ of 5. Each 50 μL sample was then diluted with 125 μL of a deionized water solution containing 50% v/v acetonitrile and 0.1% v/v trifluoroacetic acid. 5 μL of each sample was then mixed with 5 μL of a matrix solution comprising 10 mg sinapinic acid (Sigma-Aldrich, UK) dissolved in 1 mL of 50% v/v acetonitrile and 0.1% v/v trifluoroacetic acid solution. 2 μL of this mixture was then spotted in triplicate at random locations on a MALDI-MS stainless steel target and dried for 1 h at room temperature.

Samples were analyzed using a MALDI-TOF mass spectrometer (AXIMA-CRF*plus*; Shimadzu Biotech, Manchester, UK) equipped with a nitrogen pulsed 337 nm UV laser and positive ion source operated in the linear mode. A laser power of 90 mV was used and each spot was analysed using a random raster of 300 profiles with each profile containing data from five laser shots. Spectra were acquired over the mass range 1000–12000 Da with a resolution of 4000 FWHM.

Prior to analysis, mass spectra were imported into MATLAB 2008a (The MathWorks Inc., Natwick, US). Baseline correction was performed using asymmetric least squares [44] and spectra were normalized by dividing each spectrum by the square root of the sum of the squares of the spectrum [45]. PC-DFA was performed on the MALDI-TOF mass spectra as described earlier for FT-IR spectroscopy. Thirty PCs were extracted during PCA which represented 99% of the total variance in spectra from both the 12 Gy and 95 Gy treatments (including respective control treatments). As before, these PCs were passed to the DFA algorithm and Euclidean distances between control and irradiated cluster centres of each growth phase were computed.

Prior to assignment of mass peaks, mass drift in spectra was corrected using an ‘interval correlation optimized shifting’ alignment algorithm (‘icoshift’; toolbox publicly available at [46]) [47,48]. Peaks were re-aligned using a median averaging function with a maximum allowed shift of 500 Da. Subtraction spectra were then obtained by subtracting the mean spectrum of the control samples from the mean of the irradiated samples. The masses of peaks showing discrimination were tentatively assigned to proteins by reference to the UniProt Knowledgebase (Swiss-Prot and TrEMBL) [49] accessed via the ExPASy Bioinformatics Resource Portal at http://web.expasy.org/tagident [50] (accessed November 2014).

### Reduction of Fe(III) by irradiated cultures

*S*. *oneidensis* MR-1 was grown aerobically in tryptic soy broth in Erlenmeyer flasks at 30°C and shaken at 130 rpm. Late log—early stationary phase biomass was harvested by centrifugation at 4920 *g* for 20 min at 4°C and, then, washed twice in sterile 30 mM sodium bicarbonate buffer. These cell suspensions were then irradiated with 50 Gy X-radiation and cell viability was determined as described earlier. After irradiation, cell suspensions were sparged for 10 min in an 80:20 gas mix of N2:CO2. Aliquots of the irradiated cell suspension (0.2 mL) were then added to 10 mL anaerobic 30 mM bicarbonate buffer containing 20 mM lactate and 50 mM Fe(III) present as amorphous hydrous ferric oxide. Riboflavin (10 μM) was added as an electron shuttle where necessary. Triplicate experiments, along with sterile controls, were incubated in the dark at 30°C.

Each experimental bottle was shaken and then sampled periodically (anaerobically and aseptically) for 0.5 N HCl extractable Fe(II) with Fe concentrations determined by ferrozine assay with the absorbance at 562 nm measured using a UV/Vis spectrophotometer (Jenway) [51,52].

## Results and Discussion

### Growth and survival of *S*. *oneidensis* after irradiation

**Fig 1:**
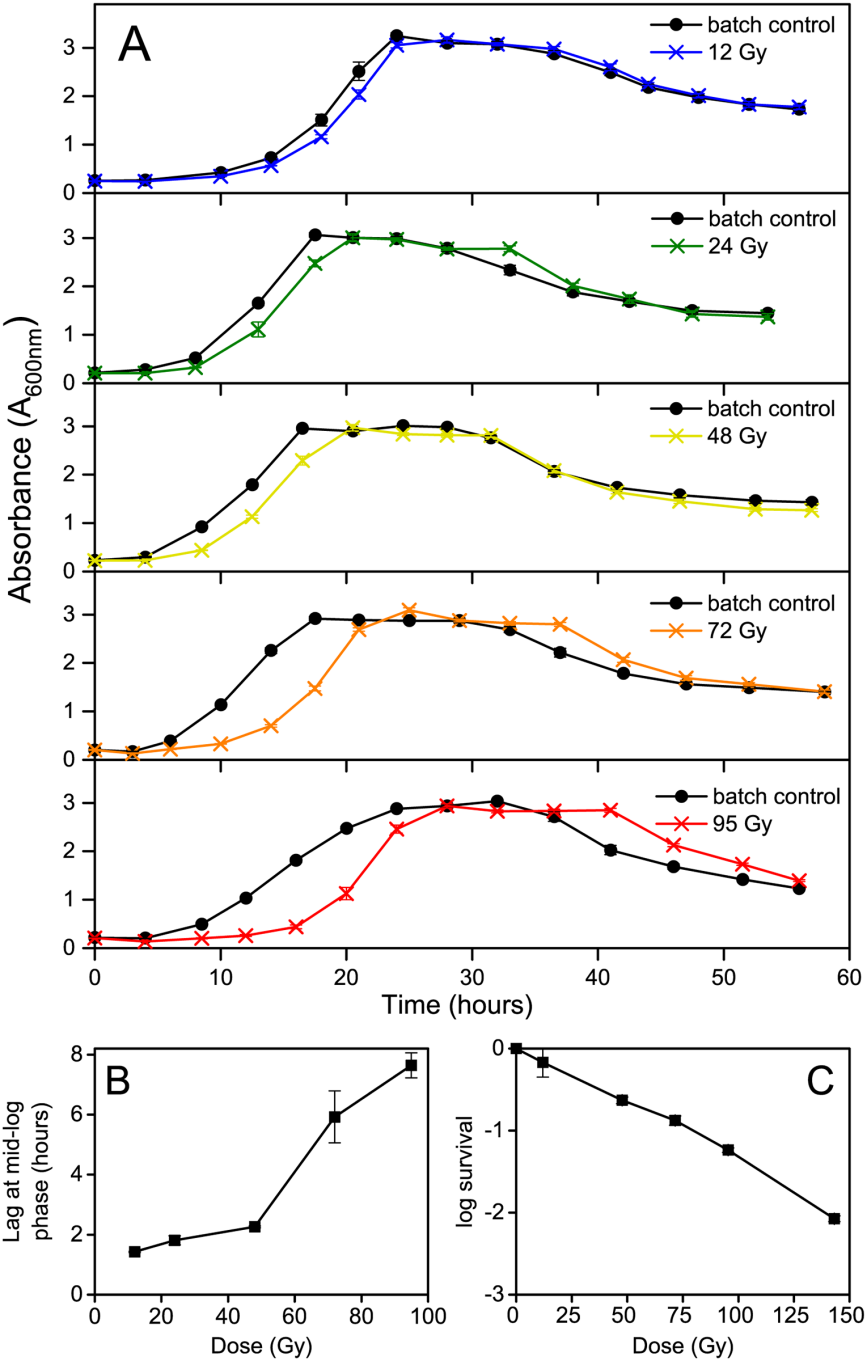
Growth, survival and extension of lag phase in X-irradiated cultures of *S*. *oneidensis* MR-1. (A) Growth profiles of aerobic cultures of *S*. *oneidensis* MR-1 (30°C) after exposure to 12, 24, 48, 72 and 95 Gy X-radiation (0.79 Gy min^-1^). Irradiations began at t = 0. A minimal growth medium was used, based on that described previously [32]. Data points show mean of triplicate batch cultures and error bars depict 95% confidence intervals. (B) Mean time difference in lag phase duration between irradiated cultures and respective controls (measured at mid exponential phase). Error bars depict 95% confidence intervals from three biological replicates. (C) Survival of *S*. *oneidensis* MR-1 exposed to acute doses of X-radiation. Cultures were irradiated in the growth medium described above, serially diluted in phosphate buffered saline and plated on to solid growth medium (same as above with 1.5% agar). Error bars depict standard error of the mean CFU mL^-1^.

Prior to analysis of metabolism, the impact of acute doses of X-radiation on the growth and viability of *S*. *oneidensis* MR-1 was assessed. The dose yielding 10% CFU survival (D_10_) was ~84 Gy and D_20_ (20% survival) was ~57 Gy (Fig 1C). These values are higher than those observed in previous studies using a ^60^Co gamma source: D_10_ = 70 Gy [21] and D_20_ = 40 Gy [23]. This is perhaps not surprising as ^60^Co gamma photons have a typical energy of 1.3 MeV compared to a maximum X-ray energy of 160 keV generated from the irradiator used in the present study. Despite this, the survival rates observed here are still indicative of the lethal effects of ionizing radiation and X-radiation is a good analogue for studying these effects.

The most marked effect of irradiation on the growth of the cultures was an extended lag phase, which increased up to ~7.5 h with a dose of 95 Gy (Fig 1A and S1 Fig). As ionizing radiation is potentially lethal, an extension of the lag phase in irradiated cultures is likely explained by a reduction in the initial active biomass immediately after irradiation (Fig 1C). However, despite receiving doses which yielded less than 10% survival (Fig 1C), ionizing radiation had no significant effect on total biomass yield (Fig 1A), suggesting that cultures were able to recover.

### Post irradiation metabolism

**Fig 2:**
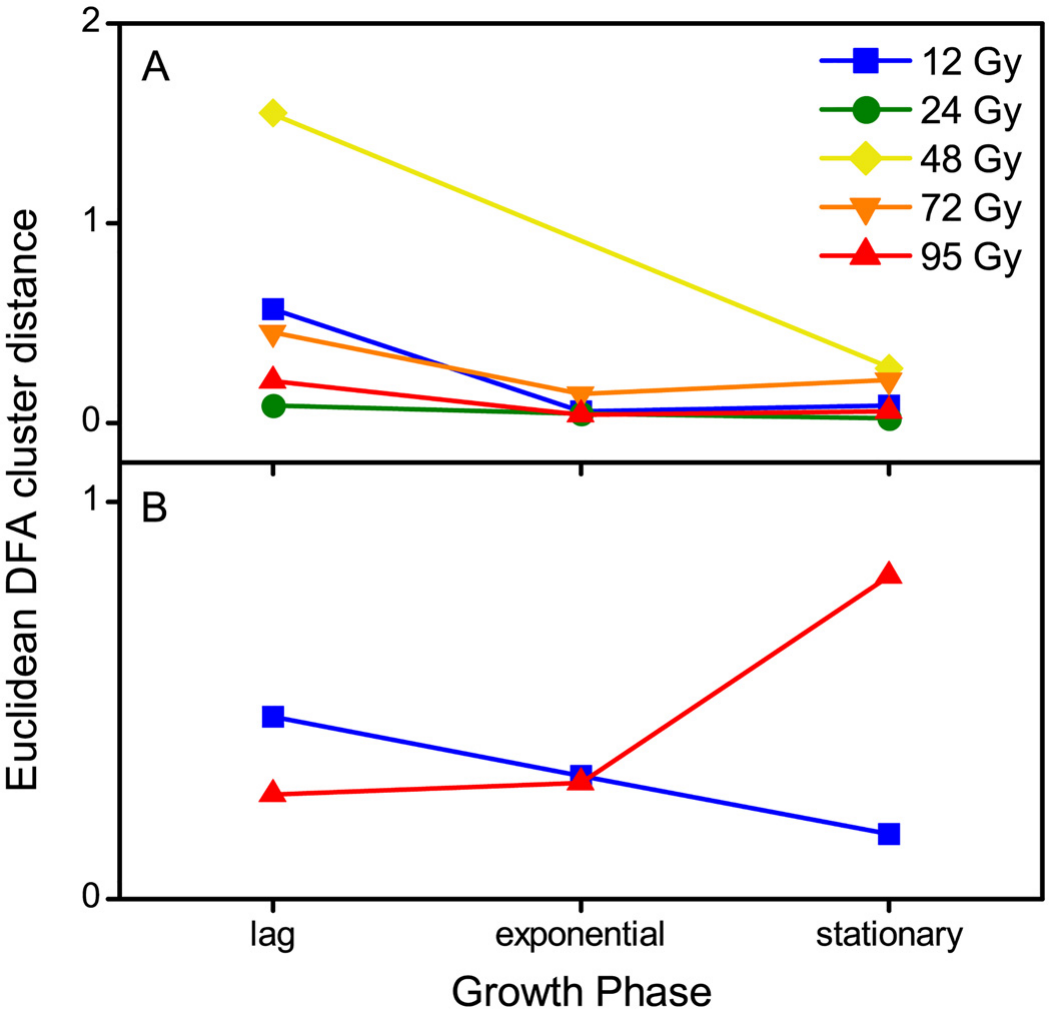
Cluster analysis of spectra from irradiated and control cultures of *S*. *oneidensis* MR-1. Euclidean distances between cluster centres of control and irradiated sample discriminant function scores extracted during principal component-discriminant function analysis (PC-DFA) applied to (A) FT-IR spectra and (B) MALDI mass spectra of lag, exponential and stationary phase X-irradiated and control cultures. Principal components 1 to 5 (FT-IR) and 1 to 30 (MALDI-MS) were used by the DFA algorithm with *a priori* knowledge of machine replicates, i.e. 1 class per sample point and treatment, giving 6 classes in total for each dose.

To assess the impact of radiation on the metabolism of *S*. *oneidensis*, samples were collected from control and irradiated cultures immediately after irradiation (lag phase), at mid exponential phase and at stationary phase and were analysed by FT-IR spectroscopy. The FT-IR spectra of control and irradiated cultures (12 to 95 Gy) were typical of metabolic fingerprints reported previously for *S*. *oneidensis* (S2 Fig) [31]. Inspection of these data by eye is limited as the spectra are qualitatively very similar. Therefore, to observe overall trends in the data and assess the importance of both dose and growth phase on the phenotype of irradiated cultures, PC-DFA was performed on FT-IR data from all growth phases for each separate dose. The Euclidean distances between the cluster centres of control and irradiated samples at each growth phase were measured and are shown in Fig 2. This showed that separation between control and irradiated sample clusters was greatest for lag phase cultures at all doses. Euclidean cluster distances decreased for all further growth phases with a slight increase in distance between the control and irradiated clusters of stationary phase cultures for some doses (12 and 72 Gy). This suggests that changes to the metabolic fingerprint of irradiated cultures are greatest during the lag phase, before the cells have time to recover.

**Fig 3:**
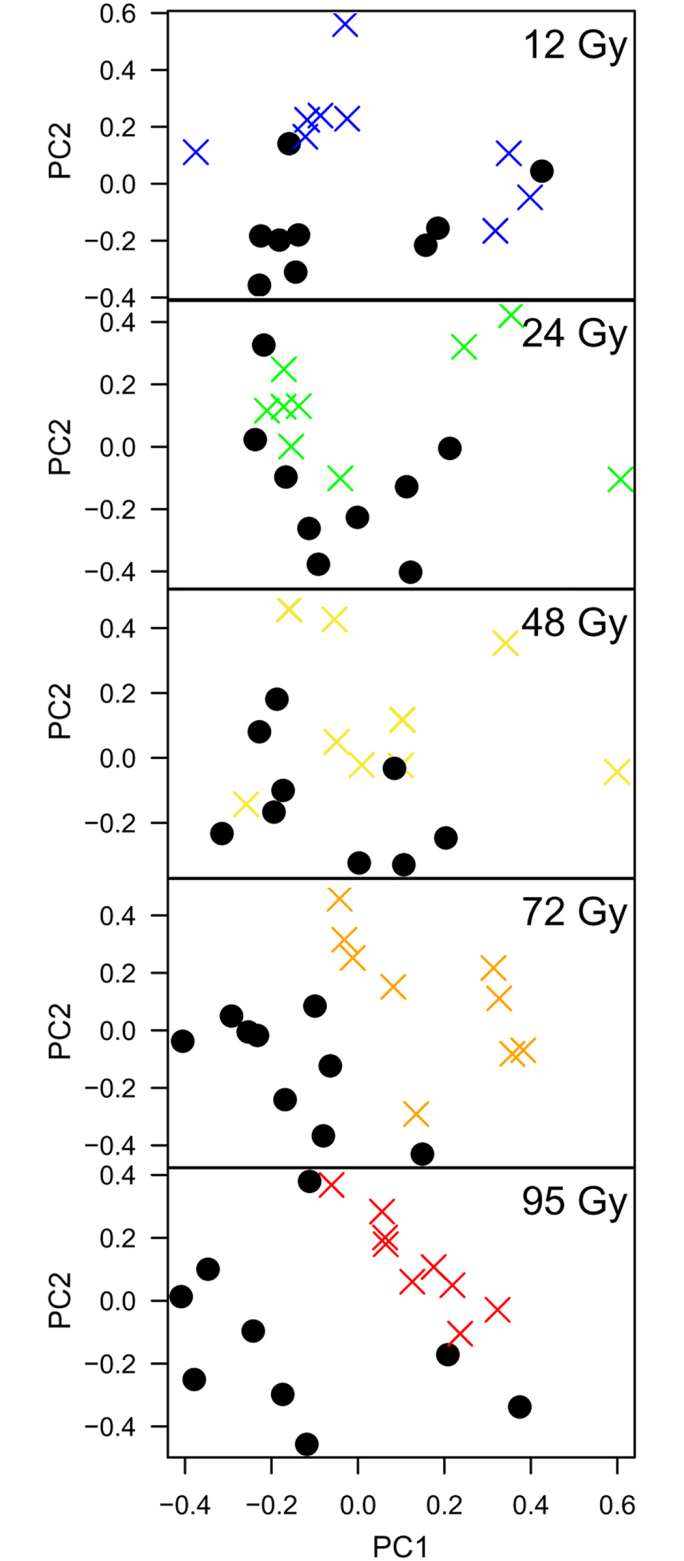
Partial least squares regression analysis of FT-IR spectra from control and irradiated cultures of *S*. *oneidensis* MR-1. Scores for the first two principal components (PC1 and PC2) extracted during partial least squares regression analysis performed on FT-IR data of control and X-irradiated cultures at lag phase. Solid black circles represent control samples and crosses represent irradiated samples. The nine replicates from each treatment are formed from three experimental replicates from each biological replicate.

PLS regression was employed to assess further the importance of dose on the metabolism of cultures in later growth phases and to determine whether an irradiated phenotype developed. PLS analysis is a supervised classification technique whereby a statistical model was supplied with information about the treatment of each sample (i.e. control or irradiated). PLS was performed on the data from all doses and the principal component scores of the lag phase data are shown (Fig 3). At doses of 12, 24, and 48 Gy, the samples of control and irradiated lag phase cultures did not display a strong degree of clustering and separation between control and irradiated samples was not observed. For samples exposed to 72 Gy and 95 Gy of X-radiation on the other hand, there was evidence of separation between control and irradiated samples, suggesting that at higher doses, there is a pronounced metabolic response to radiation. As increased energy flux at higher doses increases the frequency of ionization and damage events (both directly, via energy deposition into biomolecules, and indirectly, via the production of reactive species by water radiolysis), such dose dependent perturbation of metabolism would be expected. However, the validation plots shown in S3 Fig indicate that for all doses, except 48 Gy and 72 Gy, the *R* ^2^ values of the test samples are much lower than the model training samples, even when the number of principal components used in the model was increased. When viewed in the context of the PC-DFA data, this suggests that the changes to metabolism at lag phase may be significant but are not necessarily predictable. This is perhaps not surprising as radiation damage is indiscriminate and damage may be inflicted to a large array of cellular targets.

To assess how the recovery time after irradiation (i.e. growth phase) affected the metabolic fingerprints of cultures further, PLS was also performed on the FT-IR data from exponential and stationary phase samples. Similar to the lag phase cultures, separation between samples from control and irradiated exponential phase cultures was only observed after 72 and 95 Gy (S4 Fig). This response was not as marked as for the lag phase cultures, and again, this is somewhat reflected in the validation plots of S4 Fig. On the other hand, samples from stationary phase cultures showed a stronger degree of clustering and separation of these clusters based on their treatment (control or irradiated). Again, this effect was most evident for cultures treated with 48, 72 and 95 Gy (S5 Fig) and the ability of the PLS model to predict sample classification was best for cultures treated with 24, 72 and 95 Gy.

These observations suggest that the irradiated phenotype was preserved throughout subsequent generations of the culture despite irradiated cultures showing recovery of biomass to the same levels as non-irradiated controls (Fig 1A). Significant alteration to the regulation of a variety of genes (including metabolism related genes) measured throughout a 1 h recovery period of *S*. *oneidensis* exposed to 40 Gy irradiation has been reported previously [23]. Thus, changes to the metabolism of *S*. *oneidensis* in exponential and stationary phases observed in this study may be related to radiation induced gene regulation. Furthermore, these results suggest that such effects may be maintained throughout several doublings of the population.

### Dose related phenotypes estimated from FT-IR spectra

**Fig 4:**
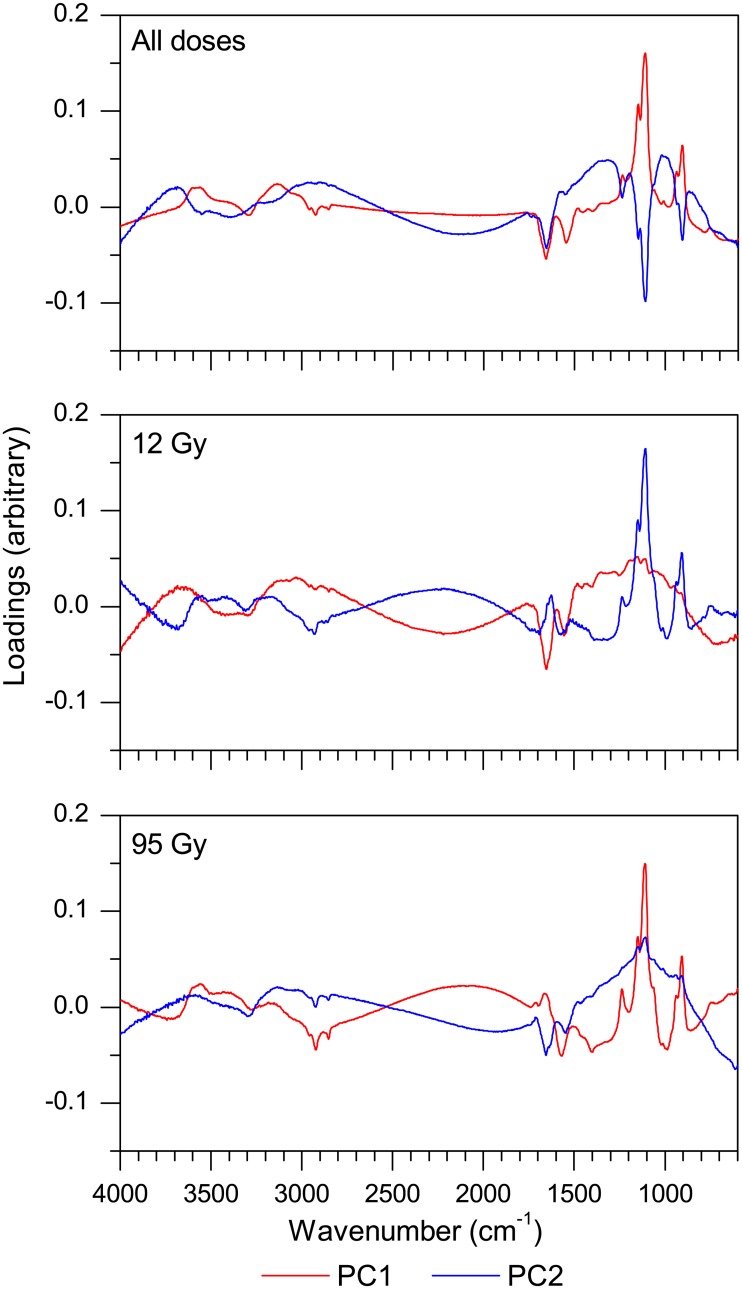
Loadings vectors from PCA analysis of FT-IR spectra of control and X-irradiated *S*. *oneidensis* MR-1. Loadings vectors for the first two principal components (PC1 and PC2) extracted during PCA applied to FT-IR spectra of lag phase cultures of *S*. *oneidensis*. Top: PCA was performed on spectra from all dose treatments (and their batch controls). PC1 = 82.2% total explained variance (TEV), PC2 = 9.0% TEV. Middle: PCA performed on spectra from 12 Gy treated cultures and batch controls. PC1 = 50.7% TEV, PC2 = 21.8% TEV. Bottom: PCA performed on spectra from 95 Gy treated cultures and batch controls. PC1 = 80.0% TEV, PC2 = 14.2% TEV.

Loadings vectors extracted during PCA and PLS analysis of IR spectra of lag phase cultures immediately after irradiation (Fig 4 and S6 Fig) highlight regions which may contribute to the separation of treated and control cultures observed in scores plots. To investigate this further, peak intensities in these regions were calculated in order to assess radiation induced changes to key molecules, which may have contributed to observed growth effects in later populations.

The region from 860 to 980 cm^-1^ was highlighted when the loadings for PCs, extracted during PCA and PLS analysis of all dose treatments, were plotted against IR wavenumber, and also from PCA applied to 12 Gy and 95 Gy treatments individually (Fig 4 and S6 Fig). This mixed spectral region has previously been assigned to carboxylic acids (941 to 950 cm^-1^) and nucleic acid related vibrations (866 to 963 cm^-1^) [31]. Three individual peaks within this region can be observed in the IR spectra: 915, 940 and 966 cm^-1^ (data not shown). The peak at 915 cm^-1^ had the greatest intensity in this region of loading plots, with the peak at 940 cm^-1^ appearing as a peak shoulder and the peak at 966 cm^-1^ not appearing at all. This suggests that wavenumbers at the lower end of this region contributed most during PCA and PLS analysis, supporting our hypothesis that changes in nucleic acid levels most likely led to this region being highlighted. Peak intensities measured at 915 cm^-1^ indicated that treatment with X-radiation led to a decrease in absorbance at all doses, except at 24 Gy, which showed no change (*p* = 0.973; *n* = 9). 12 Gy led to a decrease of 12% (*p* = 0.002; *n* = 9) whilst 95 Gy led to a decrease of 17.5% (*p* = 0.005; *n* = 9). This observation is potentially a result of radiation damage to nucleic acids, however, previous studies have indicated that whilst approximately 80% of *S*. *oneidensis* cells are killed by 40 Gy gamma radiation, this dose yields less than 1 DNA double strand break per genome [22]. As such, it is perhaps more likely that our observation is due to limited levels of nucleic acid synthesis (i.e. mRNA levels that would be associated with a growing population) in irradiated cultures as a result of radiation stress. In either case, the result is likely associated with the reduction in viability of irradiated cultures and the dose dependent increase in lag phase duration.

Loadings plots also revealed changes to regions associated with lipid related vibrations. A dose dependent increase in the CH_2_/CH_3_ asymmetric stretch intensity (2885 to 2945 cm^−1^) was observed after irradiation and was significant at doses of 48, 72 and 95 Gy (*p* < 1×10^−5^; *n* = 9). Similarly, increases in the aliphatic CH vibration (2949 and 2993 cm^−1^) were also observed after irradiation and showed dose dependence above 24 Gy (*p* < 0.003 for 48, 72 and 95 Gy; *n* = 9). Significant oxidation of membrane lipids via addition of hydroxyl radicals may result from radiolysis of fatty acids [53]. Furthermore, these oxidation reactions may occur with the various carbon moieties in the fatty acid chains and thus, changes to the CH_x_ levels may be related to hydrogen abstraction [27]. However, an increase in all three of these bond groups would unlikely be generated by oxidation reactions. Thus, these changes may not be related to specific damage processes but rather to an increase in lipid metabolism throughout irradiation, although in a previous study, genes known to be involved in lipid metabolism were down regulated in *S*. *oneidensis* as a result of radiation [23]. Whilst the exact reason for our observations remains unclear, as these functional groups are associated with membrane phospholipids, such radiation induced increases could potentially alter membrane composition, which would in turn play an important role in maintaining membrane function and fluidity [27].

The region with the largest magnitude in loadings plots (Fig 4 and S6 Fig) comprises three peaks: a main peak at 1110 cm^-1^, a peak at 1150 cm^-1^ and a peak shoulder at 1082 cm^-1^, visible in the loadings plot for 95 Gy treated cultures. Visual inspection of the IR spectra confirm the presence of these three peaks. In order to quantify changes to this spectral region, the area under the curve was computed for the region 988 to 1187 cm^-1^. A decrease in peak area was observed after irradiation with all doses, except 24 Gy, and was particularly significant after 12, 72 and 95 Gy (*p* < 0.02; *n* = 9). This decrease exhibited dose dependence above 48 Gy. The highest peak intensities for this region revealed a decrease after treatment with all doses and by as much as much as 17% and 24% in cultures irradiated with 48 and 95 Gy, respectively (*p* < 0.001 for both doses; *n* = 9).

Absorbance in this region has previously been attributed to phosphate (1037 to 1182 cm^−1^), namely the symmetric PO_2_^−^ stretch from nucleic acids, with an approximate maxima at 1106 cm^−1^ [31,54]. A decrease in nucleic acid-associated phosphate vibrations in this IR region is in good agreement with our previous observation of a decrease in the absorption in the region of 860 to 980 cm^−1^ and supports our hypothesis of radiation damage to nucleic acids and/or limited levels of nucleic acid synthesis due to radiation stress.

In addition, absorbance in the region 900 to 1200 cm^−1^ has also been assigned to saccharides, namely the C-O vibration [31,55–57]. Thus, a decrease in absorbance in this region of the IR spectra of irradiated cultures may also be attributable to lower levels of saccharides. As with nucleic acids, it is possible that this may be a result of radiation damage, or may equally be a result of decreased saccharide metabolism. Indeed, this observation may also be directly associated with the reduction in absorbance from nucleic acid associated vibrations, i.e. due to the presence of ribose in the phosphate-sugar backbone of nucleic acids. For instance, ^•^OH is known to abstract hydrogen from deoxyribose, which may lead to opening up of the of the sugar ring [58,59].

The C-N stretch of amines also absorbs in the region 1088 to 1096 cm^−1^ [60]. Amine groups have been shown to incur reaction with oxygen free radicals generated during radiolysis, with oxidation leading to the formation of α-ketoacids, aldehydes or carboxylic acids [61]. In all these mechanisms, loss of the amine group was observed and this may account for the observed decrease in absorbance at this region. Furthermore, carboxyl groups also absorb in a similar IR region [62] and this amino acid moiety has also been shown to be susceptible to free radical mediated oxidation [63]. These reactions may be related to extensive protein oxidation observed in *S*. *oneidensis* [21] and as this can modify the redox potential of the cell, these results may have implications to downstream metabolism as well as reduced protein turnover [61,63].

It is challenging to resolve the contribution of each of these bonds to the peak areas observed in this spectral range of the loadings plots (988 to 1187 cm^-1^) and thus constrain which bond or bonds are most susceptible to radiation induced changes. However, given the emergence of three peaks in this spectral range of the loadings plots, it is possible that radiation induced decreases in all three bonds are contributing to this region of the spectra.

The loadings plots of Fig 4 and S6 Fig also highlighted two peaks in the region 1479 to 1775 cm^−1^ that can be assigned to amide I and II. The mean peak areas of this region in IR spectra show a decrease after treatment with 72 and 95 Gy, however, t-tests suggest that this decrease is not statistically significant. Despite this, the emergence of these peaks in the loadings plots suggests that variance in this region between treatments contributed significantly during PCA and PLS regression.

A peak at 1236 cm^−1^ is also highlighted in the loadings from PCA and PLS. Moreover, inspection of mean IR spectra indicated that a peak in the range 1236 to 1240 cm^−1^ showed a dose dependent decrease after irradiation. The P = O of phosphate (1240 cm^−1^) [56] and the asymmetric stretch of both PO_2_^−^ (1200 to 1270 cm^−1^) and PO_3_^2−^ (1235 cm^−1^) [31] all absorb in this region. These bands are characteristic of nucleic acids and phospholipids and hence, decreases in absorbance in this region support our earlier hypotheses of radiation induced alteration to nucleic acid and fatty acid levels, the latter with implications to membrane composition. In addition, the presence of absorption bands of amide (1188 to 1265 cm^−1^; amide III) [31] and amine (1240 cm^−1^; C-N stretching) [60] in this region also supports our previous hypothesis regarding radiation damage and/or radiation induced alteration to protein metabolism. However, though a distinct peak appears in the loading plots, the relatively small magnitude of this peak (loading value) likely provides only a small contribution to PC scores and the separation of samples observed in PLS analysis and PC-DFA distance plots.

Collectively, these observations of changes in protein and amine related vibrations may be indicative of radiation damage to proteins or may suggest differences in protein metabolism immediately after irradiation. In conjunction with the tentative assignment of the region 988 to 1187 cm^−1^ to saccharides, these data may suggest that, alongside radiation damage to specific biomolecules, saccharide and protein turnover was perturbed, possibly due to irradiated cultures switching to alternative metabolic substrates as they attempt recovery. Indeed, these protein and saccharide substrates may be derived from dead cells, released during cell lysis. Alteration to the metabolism of these molecules, along with nucleic acid and fatty acid metabolism, is likely responsible for the observed growth effects and the phenotypic differences between irradiated and control cultures in subsequent generations, as discussed earlier.

### Radiation induced changes to proteins

**Fig 5:**
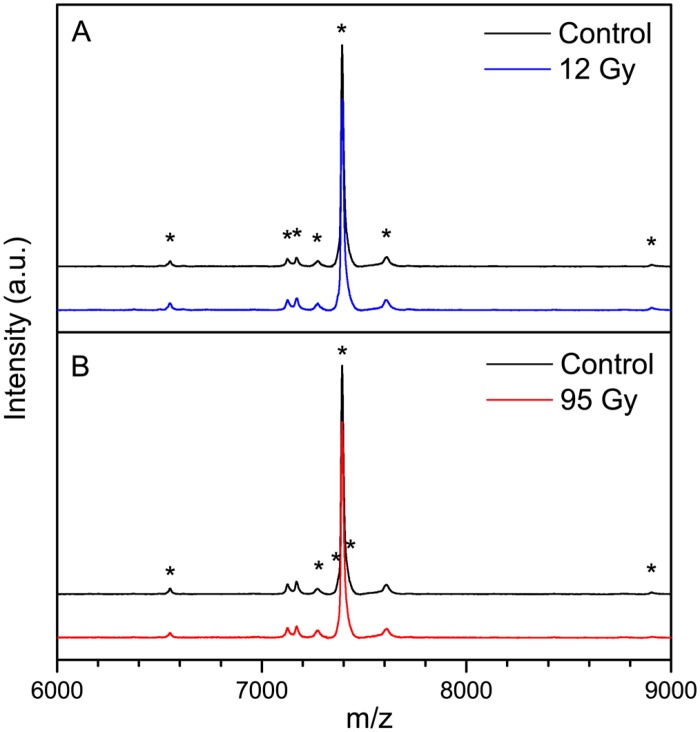
Radiation induced changes to proteins in cultures of X-irradiated *S*. *oneidensis* MR-1. Mean MALDI-MS spectra of *S*. *oneidensis* MR-1 exposed to (A) 12 Gy X-radiation and (B) 95 Gy X-radiation. Spectra have been offset in the *y*-axis so that spectral features are clearly visible. Asterisks (*) show mass peaks in irradiated spectra that are discriminant with respect to batch controls, as observed in difference spectra (mean irradiated spectrum minus mean control spectrum) (S7 Fig). The mass range has been limited in the figure to only include peaks which are discriminant and to which tentative annotations can be assigned, displayed in Table 1.

In order to examine further the potential influence of radiation on proteins of *S*. *oneidensis* revealed by FT-IR spectra and to determine whether ionizing radiation targets specific proteins, whole cell samples were analysed using MALDI-TOF-MS. Mass spectra of irradiated samples (12 and 95 Gy) and batch controls are shown in Fig 5 and peaks which appeared discriminant in subtraction spectra (S7 Fig) are highlighted. Annotations of peaks are documented in Table 1 along with tentative assignments to proteins.

**Table 1:** Anotations from UniprotKB/Swiss-Prot and UniProtKB/TrEMBL protein sequence databases of protein peaks identified in MALDI-MS difference spectra of irradiated and control cultures of *S*. *oneidensis* (S7 Fig).

Dose	Peak *m/z* (Da)	Peak range (Da)	Peak intensity relative to non-irradiated control	Protein annotations
12 Gy	6552	6547–6554	+	No match
	7125	7123–7128	+	7125 Da carbon storage regulator homolog (csrA) inferred from homology (Locus SO3426).
	7170	7168–7173	+	7170 Da 50S ribosomal protein L29 (rpmC) inferred from homology (Locus SO0239).
	7269	7266–7274	+	7269 Da uncharacterised protein (predicted; Locus SO0886); 7263 Da Molybdenum-pterin binding protein (MopI) (predicted; Locus SO1799).
	7393	7392–7393	-	7392 Da uncharacterised protein (predicted; Locus SO3548).
	7603	7601–7607	+	7603 Da uncharacterised lambda phage protein (predicted; Locus SO4795).
	8903	8899–8905	+	8901 Da protein with c-terminal DUF1078 domain (predicted; Locus SO4782).
95 Gy	6552	6547–6555	-	No match
	7269	7266–7274	+	7269 Da uncharacterised protein (predicted; Locus SO0886).
	7366	7361–7370	-	7365 Da uncharacterised protein (predicted; Locus SO4740); 7368 Da uncharacterised lambda phage protein (predicted; Locus SO3010); 7368 Da DUF3012 domain-containing lipoprotein (predicted; Locus SO0515).
	7393	7392–7394	-	7392 Da uncharacterised protein (predicted; Locus SO3548).
	7406	Peak inferred from S7 Fig	+	7406 Da cold shock protein (Csp family) inferred from homology (Locus SO1648); 7405 Da sulphur carrier protein ThiS (predicted; Locus SO2442).
	8903	8898–8903	-	8901 Da protein with c-terminal DUF1078 domain (predicted; Locus SO4782).

Many of the proteins are predicted from the *S*. *oneidensis* genome [64], and thus, do not take into account mass variability arising during translation and from processes such as post translational modification, although the latter effect is likely minimal in a bacterium. Indeed, it is also possible that radiation may modify the protein mass via fragmentation, as a result of peptide bond cleavage [61,63,65] and by ionization, which may generate subtle variations in the molecular mass. Hence, although Table 1 indicates the mass range in which the maximum peak intensities of each sample were observed, care is needed when discussing these results with regard to protein identification.

Samples exposed to 12 Gy X-radiation generally displayed peaks with a greater intensity than respective controls (S7 Fig and Table 1). These increases in protein levels as a result of irradiation may be related to the up-regulation of genes associated with amino acid transport and metabolism, and protein turnover, post translational modification and chaperones [23]. Up-regulation of proteins has also been observed in irradiated *D*. *radiodurans*, of which, some had functions related to protein turnover [66].

In addition to peak analysis, PC-DFA was applied to the data in order to assess dose dependent changes and establish whether differences in the proteome were maintained throughout growth. Euclidean PC-DFA distances show that at low dose (12 Gy), separation between control and irradiated clusters was greatest immediately after irradiation and decreasing with growth phase (Fig 2). These data suggest recovery of cultures from low dose irradiation and the up-regulation of proteins at lag phase may be related to this recovery. However, whilst a strong response of genes related to DNA repair, oxidative stress and the scavenging of reactive oxygen species has been observed during transcriptomic analysis of *S*. *oneidensis* recovering from irradiation [23], Table 1 indicates that up-regulated proteins do not appear to be related to repair mechanisms. Of the proteins that showed a change in concentration, most were unfortunately uncharacterised. An increase in a possible protein which is encoded in the *S*. *oneidensis* lambda phage was observed and this is supported by previous observations of the expression of phage genes after exposure to UV and ionizing radiation [23,67]. Indeed, the induction of phage genes and the subsequent prophage lytic cycle has been inferred to contribute to the sensitivity of this organism to ionizing radiation [23]. In addition, a putative carbon storage regulator homolog (CsrA) was observed to increase (*p* < 0.05, *n* = 9), along with a potential 50S ribosomal protein L29 (RpmC) (*p* < 0.05, *n* = 9). The reason for this response is unclear, though it may be related to a general response in metabolism and protein turnover [23] and these changes may contribute to the recovery of cultures at lower doses.

Cultures exposed to 95 Gy X-radiation showed a reduction in the levels of most detectable proteins (Fig 5, S7 Fig and Table 1), with respect to control treatments. Peaks at 7366, 7393 and 8903 Da showed a decrease as a result of radiation; however, the functions of predicted proteins at these masses are uncharacterised. This may be the result of down regulation of genes related to protein metabolism [23], however, at this higher dose, it is perhaps more likely a result of protein damage arising from reactions such as oxidation and carbonylation [21,25]. In contrast to the reduction observed in most proteins after 95 Gy irradiation, increased levels of a possible protein at 7406 Da was observed. This mass can be tentatively assigned to a cold shock protein (Csp family) or a sulfur carrier protein (ThiS). Cold shock proteins may serve as RNA chaperones and gene regulators and have various physiological roles in response to a variety of stresses [68,69]. The up-regulation of this protein may be a reaction to cellular radiation stress which may share physiological triggers with cold shock. In addition, cold shock proteins have also been implicated in regulation of membrane fluidity [70] and thus, the up-regulation of this protein could also be related to the increase in lipid related bonds revealed by FT-IR spectra. A protein with mass 7269 Da also increased, which can be tentatively assigned to a predicted protein at Locus SO0886, although its function is currently uncharacterised.

In contrast to the 12 Gy treatment, the clusters of control and 95 Gy treated samples showed greatest Euclidean PC-DFA distances between stationary phase samples (Fig 2). These data suggest that, whilst the proteome of a 12 Gy treated culture appears to show recovery, at 95 Gy, alteration to the proteome may be preserved, or indeed exacerbated through successive generations of the culture. This response is likely a result of changes in gene expression which may be persistent throughout the exponential growth of cultures irradiated to a high dose. Thus, it is evident that radiation induced changes to the levels of protein in *S*. *oneidensis* are dose dependent and this dose dependence also strongly influences the phenotype in latter growth phases.

### Radiation induced Fe(III) reduction

**Fig 6:**
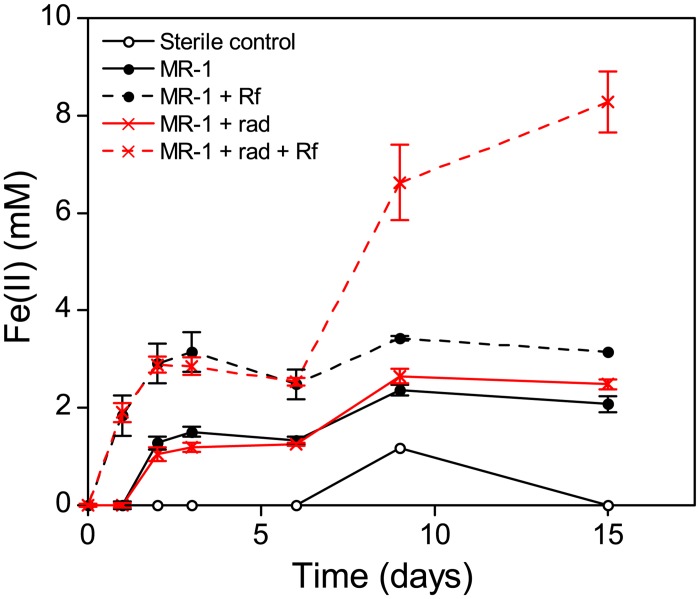
Fe(III) reduction by irradiated *S*. *oneidensis* MR-1. Cultures of *S*. *oneidensis* (MR-1) were grown aerobically in tryptic soy broth (30°C; 130 rpm) to late log–early stationary phase. Biomass was harvested and washed twice in sterile 30 mM sodium bicarbonate buffer prior to irradiation with 50 Gy X-radiation (rad). Immediately after irradiation, cell suspensions were driven anoxic with an 80:20 gas mix of N2:CO2 prior to inoculation into an anoxic medium containing 20 mM lactate as electron donor, 50 mM Fe(III) as poorly crystalline insoluble Fe(III) oxide and 30 mM sodium bicarbonate. 10 μM riboflavin (Rf) was added to media post-irradiation as an electron shuttle where necessary. Fe(II) concentrations were determined by ferrozine assay after extraction with 0.5 N HCl. Error bars depict standard error of the mean of triplicate experiments.

The data described above suggest that ionizing radiation alters protein and lipid levels. As these molecules are important components of biological membranes, radiation induced changes to these molecules could alter the integrity and function of *S*. *oneidensis* membranes, where many of the respiratory chain components and cytochromes required to respire alternative electron acceptors, such as Fe(III), are located. Hence, the ability of the irradiated phenotype to reduce poorly crystalline insoluble Fe(III) oxide was assessed. Cell cultures were irradiated with 50 Gy X-radiation, selected as an approximate median dose from the doses used in the metabolic experiments described above. Systems containing irradiated biomass displayed more than double the levels of Fe(III) reduction (Fig 6) despite biomass displaying only 2.3% viability with respect to non-irradiated controls (determined, as before, by serial dilution in PBS and CFU counts on solid minimal medium agar plates). It is evident that much of the Fe(III) in the poorly crystalline insoluble Fe(III) oxide used in this experimental system was not directly accessible for enzymatic reduction. Indeed, the extent of Fe(II) generation in the systems containing irradiated *S*. *oneidensis* was only increased with the addition of riboflavin, indicating that any radiation induced metabolic changes required the presence of an electron shuttle to facilitate reduction of Fe(III). The reason for this is unclear, although it could be related to a general up-regulation of metabolism post-irradiation, or physical damage to the cell structure facilitating extracellular electron transfer. However, the fact that this phenomenon is only observed in the presence of an electron shuttle suggests that this effect is likely not the result of the release of soluble reducing equivalents from lysed cells killed by radiation. The precise mechanism underpinning this result clearly warrants further work, ideally focusing on anaerobically grown cultures adapted to Fe(III) reducing conditions, rather than the aerobically grown cultures that were used in this experiment to facilitate comparisons with the earlier experiments described above. In addition, future experiments may be expanded to determine whether this phenomenon is limited to insoluble Fe(III) or is also applicable to chelated soluble Fe(III) and other inorganic anaerobic electron acceptors.

## Conclusions

In summary, these results suggest that ionizing radiation impacts upon the viability and growth of aerobic cultures of *S*. *oneidensis* over a range of doses. Modification to the nucleic acid, protein and lipid content of cells characterises the metabolism immediately after irradiation and multivariate statistical analyses reveal the development of an irradiated phenotype throughout multiple generations despite biomass recovery. Both fatality and metabolic changes are likely contributors to observed growth effects and this may have implications for growth in subsurface environments, where doubling times are large.

As protein and lipid levels are fundamental to the integrity and functionality of membranes, ionizing radiation may have significant implications for the long-term metabolism and hence functionality of *S*. *oneidensis* during bioremediation applications. Furthermore, such biochemical changes may also be pivotal to the control *S*. *oneidensis* may exact on the chemistry of key electron acceptors, such as Fe(III) and radionuclides such as U(VI), in the near field environment of a geological disposal facility for radioactive waste. Indeed, previous work has shown that gamma radiation may enhance the bioavailability of Fe(III) oxides, potentially stimulating an Fe(III)-reducing microbial community [71]. Hence, further work is required to characterise fully the impact of radiation on the respiratory capabilities of *S*. *oneidensis*, particularly the ability of the irradiated phenotype to respire alternative electron acceptors, such as redox active metals and radionuclides.

## Supporting Information

S1 Fig Growth profiles of *S*. *oneidensis* MR-1, plotted on a log scale, after exposure to 12, 24, 48, 72 and 95 Gy X-radiation. Data points show mean of triplicate batch cultures and error bars depict 95% confidence intervals.
(EPS)

S2 Fig FT-IR spectra of cells of lag phase *S*. *oneidensis* MR-1 cultures exposed to X-radiation. (EPS)

S3 Fig Scores for the first two principal components (PC1 and PC2) extracted during partial least squares regression analysis performed on FT-IR data of control and X-irradiated cultures at lag phase (left panel) and validation plots for each model (right panel). R2 = *R* ^*2*^, generated from cross validation of each model. Solid black circles represent control samples and crosses represent irradiated samples. The nine replicates from each treatment are formed from three experimental replicates from each biological replicate.
(TIF)

S4 Fig Scores for the first two principal components (PC1 and PC2) extracted during partial least squares regression analysis performed on FT-IR data of control and X-irradiated cultures at exponential phase (left panel) and validation plots for each model (right panel). R2 = *R* ^*2*^, generated from cross validation of each model. Solid black circles represent control samples and crosses represent irradiated samples. The nine replicates from each treatment are formed from three experimental replicates from each biological replicate.
(TIF)

S5 Fig Scores for the first two principal components (PC1 and PC2) extracted during partial least squares regression analysis performed on FT-IR data of control and X-irradiated cultures at stationary phase (left panel) and validation plots for each model (right panel). R2 = *R* ^*2*^, generated from cross validation of each model. Solid black circles represent control samples and crosses represent irradiated samples. The nine replicates from each treatment are formed from three experimental replicates from each biological replicate.
(TIF)

S6 Fig Regression co-efficients extracted during partial least squares (PLS) analysis performed on FT-IR spectra of lag phase cultures of *S*. *oneidensis*. PLS analysis was performed on spectra from all dose treatments and their batch controls.
(TIF)

S7 Fig MALDI-MS difference spectra of *S*. *oneidensis* MR-1 exposed to 12 Gy (top panel) and 95 Gy X-radiation (bottom panel). Spectra show the result of the mean irradiated spectrum minus the mean control spectrum with labels indicating the masses of peaks that show statistical significance according to, at minimum, the standard error of the mean of 9 replicate measurements. The mass range has been limited in the figure to only include peaks which show a difference and to which tentative annotations can be assigned, displayed in Table 1.
(EPS)
