# Supplementary figures and images for: Phenotypic Characterisation of Shewanella oneidensis MR-1 Exposed to X-Radiation
Source: PLoS One. 2015 Jun 22;10(6):e0131249. doi: 10.1371/journal.pone.0131249 (PMC4476702; doi:10.1371/journal.pone.0131249)

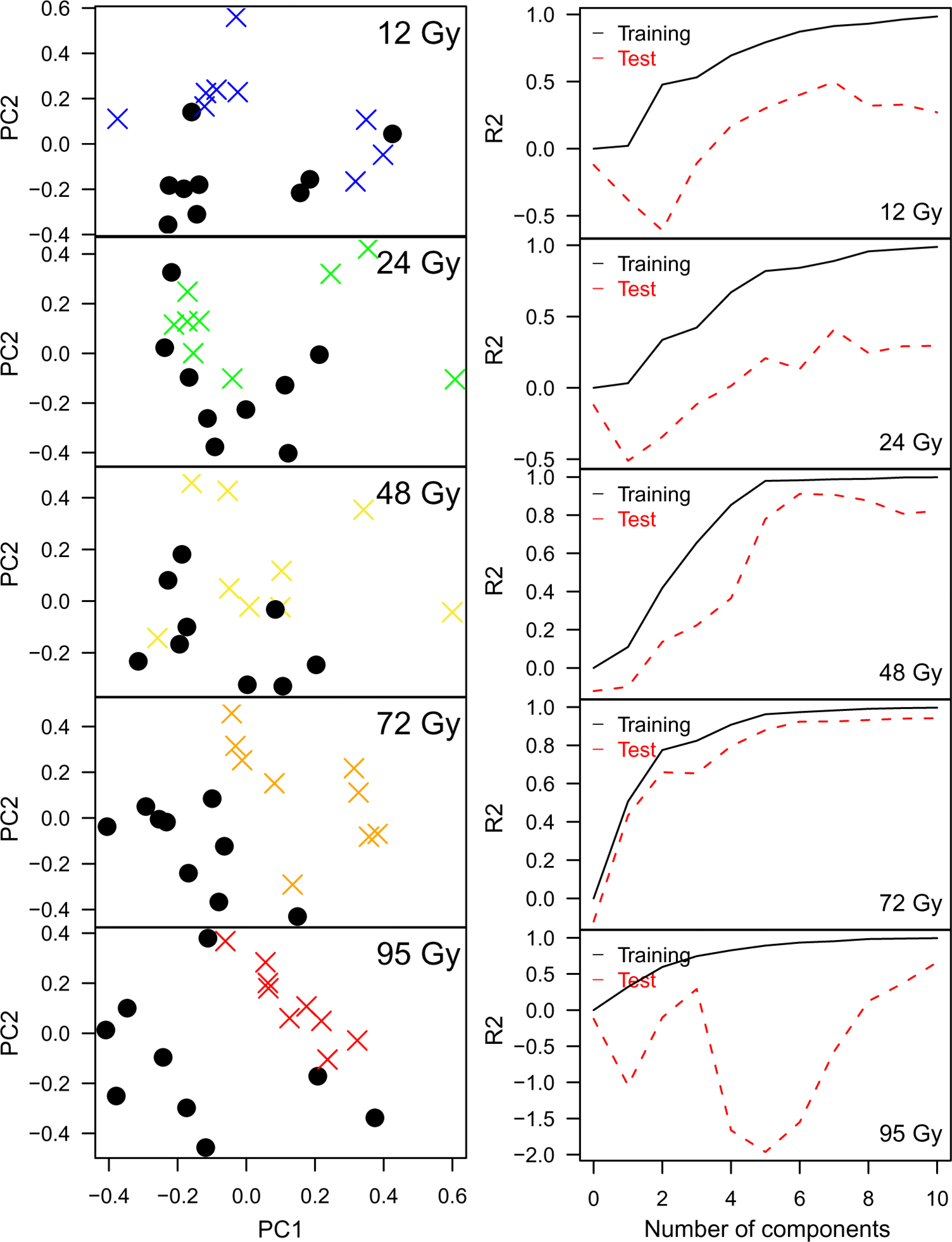

Supplement: S3 Fig — R2 = R 2, generated from cross validation of each model. Solid black circles represent control samples and crosses represent irradiated samples. The nine replicates from each treatment are formed from three experimental replicates from each biological replicate. (TIF) [file pone.0131249.s003.tif]

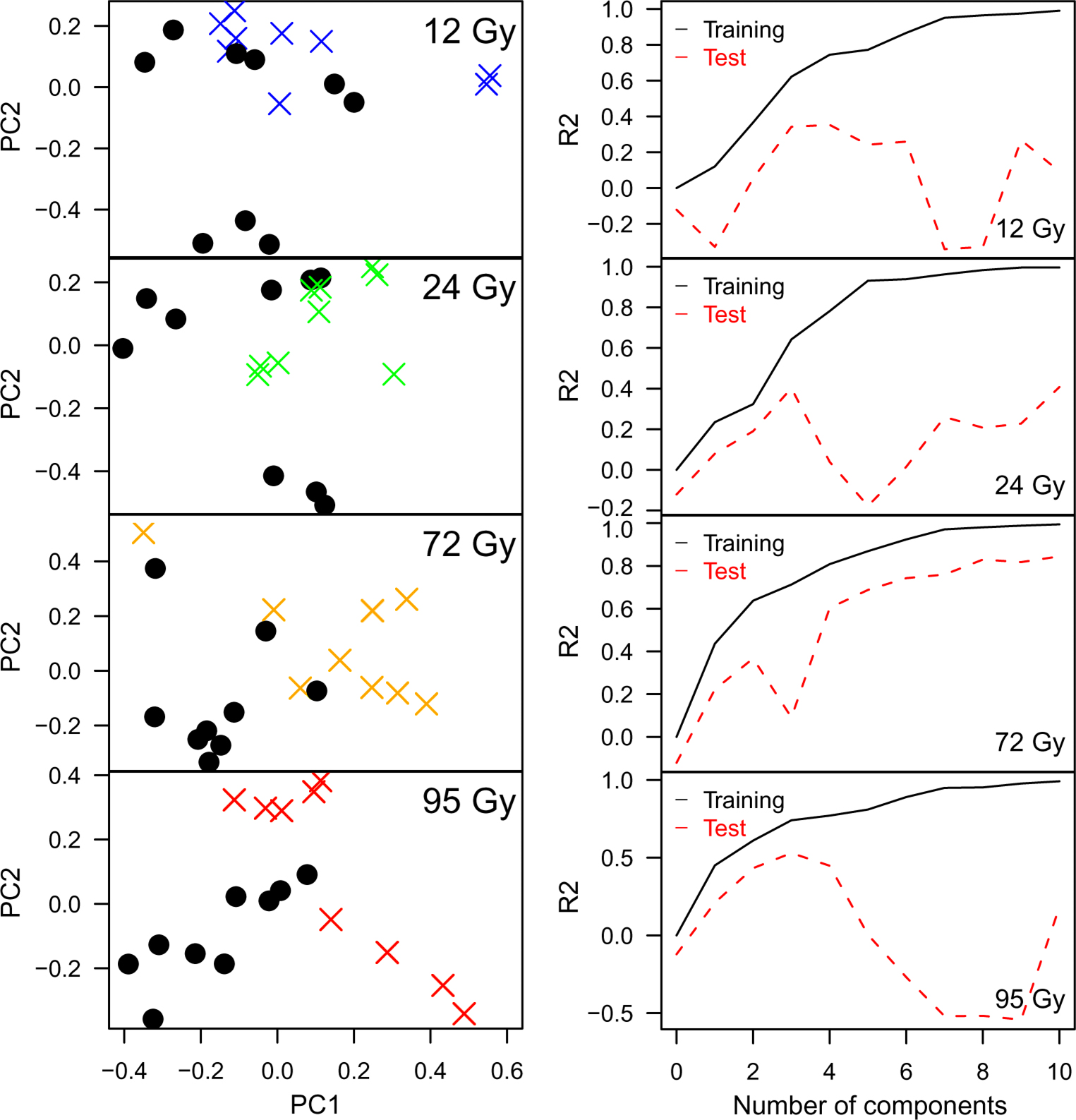

Supplement: S4 Fig — R2 = R 2, generated from cross validation of each model. Solid black circles represent control samples and crosses represent irradiated samples. The nine replicates from each treatment are formed from three experimental replicates from each biological replicate. (TIF) [file pone.0131249.s004.tif]

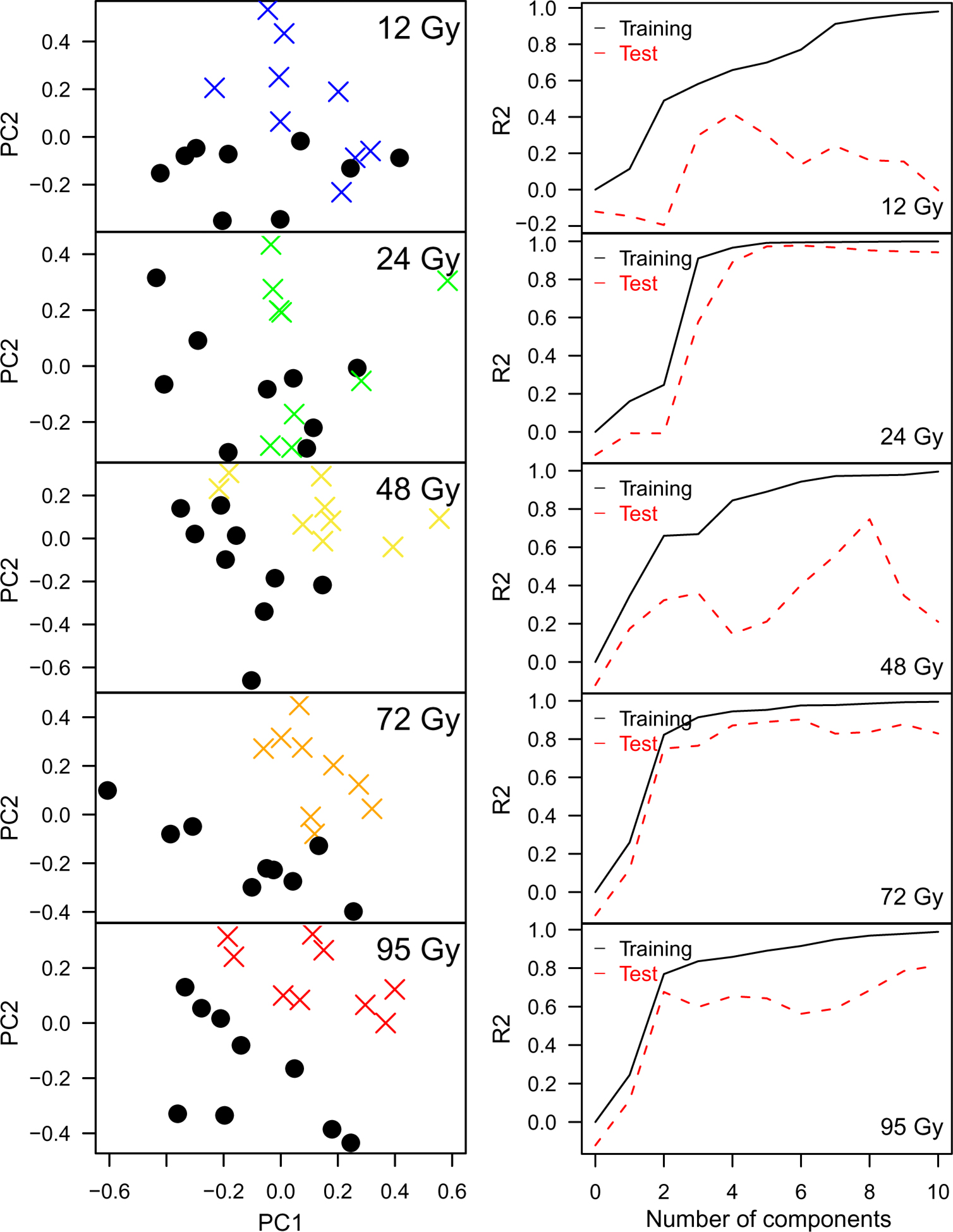

Supplement: S5 Fig — R2 = R 2, generated from cross validation of each model. Solid black circles represent control samples and crosses represent irradiated samples. The nine replicates from each treatment are formed from three experimental replicates from each biological replicate. (TIF) [file pone.0131249.s005.tif]

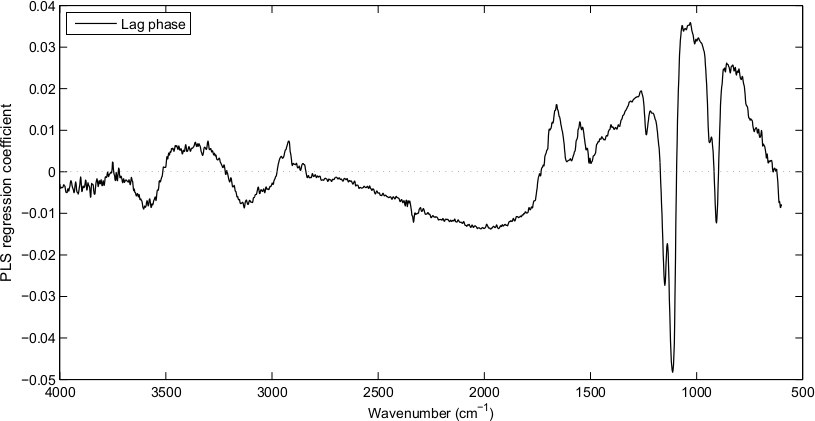

Supplement: S6 Fig — PLS analysis was performed on spectra from all dose treatments and their batch controls. (TIF) [file pone.0131249.s006.tif]
